# Phosphate of Lime

**Published:** 1871-04

**Authors:** 


### PHOSPHATE OF LIME.

We have for many years used the phosphate of lime in practice, with the view of improving the condition of the teeth, in which that constituent was deficient in quantity, and so frequent and so definite has been the results, that we are constrained to use it almost constantly. The most practicable form, has been a matter of no little solicitude. The formula should differ with varying indications; but what has interested us most was to get the best preparation of the simple phosphate. For many years we used the solid substance—the refined lime phosphate of commerce, with a consciousness, all the time, however, that a solution of it would be better. With the object of obtaining the best simple solution of the phosphate of lime, we made known our want to Mr. E. S. Wayne, a very superior chemist and pharmaceutist, who gave us the following formula:

R.—Acid phosphoric dil. U. S. P. q. s.
Phosphate lime, fresh precipitated. q. s.

Dissolve the phosphate of lime to saturation in the acid, filter, and add an equal quantity of pure glycerin, or of sugar, preferably the glycerin. (In case of anæmic condition, phosphate of iron may with advantage be added, also made tonic by the addition of sulph. quinia.)

This makes a very pleasant syrup, and one holding in solution a very large quantity of the phosphate; an ordinary tea-spoonful containing about $5\frac{1}{2}$ grains. T.

